# 3,5-Diiodo-L-Thyronine Exerts Metabolically Favorable Effects on Visceral Adipose Tissue of Rats Receiving a High-Fat Diet

**DOI:** 10.3390/nu11020278

**Published:** 2019-01-27

**Authors:** Elena Silvestri, Rosalba Senese, Federica Cioffi, Rita De Matteis, Davide Lattanzi, Assunta Lombardi, Antonia Giacco, Anna Maria Salzano, Andrea Scaloni, Michele Ceccarelli, Maria Moreno, Fernando Goglia, Antonia Lanni, Pieter de Lange

**Affiliations:** 1 Dipartimento di Scienze e Tecnologie, Università degli Studi del Sannio, via Port’Arsa 11, 82100 Benevento, Italy; silvestri@unisannio.it (E.S.); federica.cioffi@unisannio.it (F.C.); agiacco@unisannio.it (A.G.); m.ceccarelli@gmail.com (M.C.); moreno@unisannio.it (M.M.); goglia@unisannio.it (F.G.); 2 Dipartimento di Scienze e Tecnologie Ambientali, Biologiche e Farmaceutiche, Università‘ degli Studi della Campania Luigi Vanvitelli, via Vivaldi 43, 81100 Caserta, Italy; Rosalba.SENESE@unicampania.it; 3 Dip. Scienze Biomolecolari, Salute, Università di Urbino “Carlo Bo”, via Maggetti, 26, 61029 Urbino, Italy; rita.dematteis@uniurb.it (R.D.M.); davide.lattanzi@uniurb.it (D.L.); 4 Dipartimento di Biologia, Università degli Studi di Napoli “Federico II”, via Cinthia, 80126 Napoli, Italy; aslombar@unina.it; 5 Proteomics & Mass Spectrometry Laboratory, ISPAAM, National Research Council, via Argine, 1085, 80147 Naples, Italy; annamaria.salzano@cnr.it (A.M.S.); andrea.scaloni@ispaam.cnr.it (A.S.)

**Keywords:** 3,5-diiodo-L-thyronine, visceral white adipose tissue, ATGL, hormone sensitive lipase, lipolysis, proteomics

## Abstract

When administered to rats receiving a high-fat diet (HFD), 3,5-diiodo-L-thyronine (3,5-T2) [at a dose of 25 μg/100 g body weight (BW)] is known to increase energy expenditure and to prevent HFD-induced adiposity. Here, we investigated which cellular and molecular processes in visceral white adipose tissue (VAT) contributed to the beneficial effect of 3,5-T2 over time (between 1 day and 4 weeks following administration). 3,5-T2 programmed the adipocyte for lipolysis by rapidly inducing hormone sensitive lipase (HSL) phosphorylation at the protein kinase A-responsive site Ser^563^, accompanied with glycerol release at the 1-week time-point, contributing to the partial normalization of adipocyte volume with respect to control (N) animals. After two weeks, when the adipocyte volumes of HFD-3,5-T2 rats were completely normalized to those of the controls (N), 3,5-T2 consistently induced HSL phosphorylation at Ser^563^, indicative of a combined effect of 3,5-T2-induced adipose lipolysis and increasing non-adipose oxidative metabolism. VAT proteome analysis after 4 weeks of treatment revealed that 3,5-T2 significantly altered the proteomic profile of HFD rats and produced a marked pro-angiogenic action. This was associated with a reduced representation of proteins involved in lipid storage or related to response to oxidative stress, and a normalization of the levels of those involved in lipogenesis-associated mitochondrial function. In conclusion, the prevention of VAT mass-gain by 3,5-T2 occurred through different molecular pathways that, together with the previously reported stimulation of resting metabolism and liver fatty acid oxidation, are associated with an anti adipogenic/lipogenic potential and positively impact on tissue health.

## 1. Introduction

The reduced life expectancy of obese subjects is due to a health-threatening accumulation of body fat, which is generally associated with insulin resistance and cardiovascular abnormalities [1,2]. Among adipose tissues, visceral depots are regarded as the major cause of these alterations [3]; thus, understanding the development, function and manipulation of visceral adipose tissue is becoming an urgent need. White adipose tissue (WAT) is physiologically important for transport, synthesis, storage and mobilization of lipids as well as for insulin-dependent glucose uptake and endocrine control of energy homeostasis [4]. Enlargements of visceral adipose tissue (VAT) mass, which is strongly influenced by diet and is linked to a greater susceptibility of developing obesity-related pathologies [5], encompass an increase in both adipocyte cell size (hypertrophy) and cell number (hyperplasia), leading to dysfunction of adipocytes. Several lines of evidence suggest that VAT from obese humans and rodent models of obesity shows inflammation, is poorly oxygenated and is exposed to oxidative conditions; thus, factors involved in inflammation, hypoxia and oxidative stress are considered to be a part of the pathology causing adipocyte dysfunction [6]. Although hardly addressed, mitochondria play an important role in WAT physiology and pathology. Indeed, WAT mitochondria are crucial to adipogenic differentiation [7] as well as to adipokine secretion [8,9]. Excessive nutrient intake may lead to WAT mitochondrial dysfunction, with impaired substrate oxidation, deleterious effects on lipid and glucose metabolism [10], and production of reactive oxygen species (ROS), thereby accentuating the development of obesity and associated disorders [11,12]. 

Thyroid hormones are important regulators of WAT function. In this context, 3,3’,5-triiodo-L-thyronine (T3) regulates several genes involved in lipolysis, lipogenesis, thermogenesis, mitochondrial function and nutrient availability [13]. T3 also acts as a significant determinant in the conversion of preadipocytes to adipocytes [14]. However, given the fact that T3 induces heart rhythm abnormalities, applying this hormone as a pharmacological compound has been discouraged [15]. 

Recently, the thyroid hormone metabolite 3,5-diiodo-L-thyronine (3,5-T2) has been shown to exhibit specific metabolic activities whose characterization would provide novel information on thyroid control of metabolism both in physiological and pathological contexts, and would help to identify novel mediators, pathways and targets of thyroid hormone action. When exogenously administered to rodents, 3,5-T2 rapidly increases the resting metabolic rate [16,17] and prevents diet-induced overweight and insulin resistance [18,19,20,21]. Few studies have evaluated cellular and molecular effects of endogenous and exogenous 3,5-T2 in humans [22,23,24,25,26]. While several lines of evidence suggest that 3,5-T2 seems to act through nuclear thyroid hormone receptors (THRs)-independent pathways, with mitochondria as a likely cellular target [27], THR-dependent actions have also been described [28,29]. However, the cellular and molecular mechanisms through which 3,5-T2 elicits its multiplicity of actions are incompletely understood; this is complicated by the fact that thyroid metabolite activities appear to be dose-, species-, diet- and age-specific [17,30,31,32,33,34]. We previously reported that 3,5-T2, when administered (at the dose of 25 μg/100 g BW) to high fat diet (HFD) fed rats for 4 weeks, can prevent body adiposity and insulin resistance without inducing deleterious side effects, i.e., alterations of the hypothalamus-pituitary-thyroid (HPT) axis, and of skeletal muscle and heart mass [18,19,20,21,35]. However, administration of higher doses of 3,5-T2 (75–1250 μg/100 g) to rodents suggested a possible interference of 3,5-T2 on the corresponding HPT axis and heart [31,36,37]. It is worth mentioning that 3,5-T2 did not seem to suppress TSH as strongly as T3 [38], and cardiac readouts tentatively represented a physiological adaptation to increased metabolic rate, perhaps implying potential for separation of desirable from thyrotoxic effects.

Histological analyses of VAT revealed that 3,5-T2 administration to HFD rats can prevent VAT hypertrophy [19], but the underlying biochemical pathways and related molecular processes remain unknown. In this study, we investigated cellular and molecular mechanisms responsible for VAT phenotypic changes after administration of 3,5-T2 to HFD rats. We performed a time-course analysis (spanning 2 weeks) of the effects of 3,5-T2 administration on body weight gain, visceral adiposity, adipocyte hyperplasia and hypertrophy, and lipolytic pathways combined with a comparative proteomic analysis of VAT from HFD, 3,5-T2-treated HFD (HFD-T2) and control (N) rats, at the 4-week time-point. Using this approach, potential molecular mechanisms underlying the observed phenotypic changes induced by 3,5-T2 in VAT were identified. Overall, these data contribute to providing new clues for the metabolic effects of 3,5-T2, expanding the view obtained by our previous investigations on metabolic phenotypes, i.e., improvement of insulin sensitivity and adiposity.

## 2. Materials and Methods

### 2.1. Materials

All solvents used were of liquid chromatography-mass spectrometry (LC-MS) grade from Sigma-Aldrich (St. Louis, MO, USA) and Carlo Erba (Milan, Italy). Immobilized pH-gradient (IPG) and ampholites were purchased from Bio-Rad (Hercules, CA, USA). 3,5-T2, 3-((3-cholamidopropyl) dimethylammonio)-1-propanesulfonate (CHAPS), dithiothreitol (DTT), tosyl-phenylalanyl chloromethyl ketone-treated porcine trypsin, and other reagents for electrophoresis under native and denaturant conditions, or histochemical staining of respiratory complex activity were from Sigma-Aldrich. µZipTip C18 devices were from Millipore (Bedford, MA, USA).

### 2.2. Animals

Animal care and experiments were conducted in accord with the guidelines of the Ethics Committee of the University of Campania “Luigi Vanvitelli”. Male Wistar rats (250–300 g) (Charles River, Lecco, Italy) were kept one per cage in a temperature-controlled room, at 28 °C, under a 12-h light and 12-h dark cycle. Water was available *ad libitum*. Rats were divided into 3 groups (6 animals/group). The first group (N) received a standard chow diet [Fatty acid content (mg/kg): palmitate (16:0) 4387; palmitoleate (16:1) 202; stearic (18:0) 675; oleic (18:1) 5046; linoleic (18:2) 12335; linoenic (18:3) 1169. Total metabolizable percentage of energy: carbohydrates 60.4; proteins 29; fat 10.6 J/J; 15.88 KJ gross energy/g (Muscedola s.r.l., Milan, Italy)] with a daily injection of vehicle (10 mM NaOH dissolved in physiological buffer). The second group (HFD) received a high-fat diet (consisting of 280 g chow diet supplemented with 395 g of lyophilized lamb meat (Liomellin, Milan, Italy), 120 g cellulose (Sigma-Aldrich, Milano, Italy), 20 g mineral mix (ICN biomedical, Solon, OH, USA), 7 g vitamin mix (ICN) and 200 g low-salt butter (Lurpak^®^, Denmark) with a daily injection of vehicle. The third group (HFD-T2) received the same high-fat diet with a daily i.p. injection of 3,5-T2 (Sigma Aldrich, Milano, Italy) (25 µg/100 g BW) [17,18,19,20]. Animals were killed at four-time points: 1 day, 1 week, 2 weeks, and 4 weeks following treatment. In agreement with previous observations [18], neither HFD nor 3,5-T2-treatment altered the basal TSH values of the treated animals (data not shown). At the end of the treatments, rats were anesthetized by intraperitoneal injection of chloral hydrate (40 mg/100 g BW), then killed by decapitation. Visceral mesenteric white adipose tissue (VAT) was excised, weighed, and either immediately processed for isolation of fresh adipocytes or for histochemical analysis, or immediately frozen in liquid nitrogen and stored at −80 °C for later processing. The minimum sample size (*n* = 4) was calculated based on a G* Power Test, which was performed using software obtained from the University of Dusseldorf (http://www.gpower.hhu.de/). The power was 0.95, the effect size (f) was 2.26 and the α was 0.05.

### 2.3. Histological Analysis, Adipocyte Size Determination and BS-1 Staining

Samples of VAT were fixed by immersion in 4% formaldehyde in 0.1 M phosphate buffer, overnight, 4 °C. The samples were dehydrated in ethanol, cleared, and embedded in paraffin blocks. Tissues were cut into serial 6-πm-thick sections, and stained with hematoxylin-eosin for morphological examination. For adipocyte size quantification, evaluations were performed on 3 different hematoxylin-eosin slides (sections every 400 µm) for each animal and at least 400 adipocytes per animal were analyzed. Sections were viewed with a Nikon Eclipse 80i light microscope (Nikon Instruments, Milan, Italy) at 20× magnification. Images were obtained with a DS-5M camera (Sony, Italy) connected to an ACT-2U image analyzer (Nikon, Italy). The mean surface area and the frequency distribution were calculated from at least 4 rats for each group; adipocyte size distribution is presented as percentage of the total amount of cells. Vascularization of VAT was assessed by use of the biotinylated forms of *Bandeiraea simplicifolia* agglutinin 1 (BS-1) (Sigma-Aldrich, Milano, Italy, BS-1; L3759), which is an immunohistochemical marker of endothelial cells, as previously described [39]. For detection, a Vectastain avidin-biotin complex- alkaline phosphatase (ABC-AP) Kit (Vector Lab, Burlingame, CA, USA) was used, followed by development with a Fucsin + Substrate Chromogen System (Dako, Agilent, Santa Clara, CA, USA).

### 2.4. Preparation of Total Tissue Lysates

VAT was homogenized in lysis buffer containing 20 mM Tris-HCl, pH 7.5, 150 mM NaCl, 1 mM EDTA, 1 mM EGTA, 2.5 mM Na_2_H_2_P_2_O_7_, 1 mM b-CH_3_H_7_O_6_PNa_2_, 1 mM Na_3_VO_4_, 1 mM PMSF, 1 mg/mL leupeptin, and 1% Triton X-100 (all from Sigma-Aldrich, Milano, Italy) using an ultraturrax, and centrifuged with a Optima TLX instrument (Beckman Coulter, Milan, Italy) at 16,000 × *g*, for 10 min, at 4 °C. Supernatants were ultracentrifuged at 100,000 × *g*, for 10 min, at 4 °C. Protein concentration of the ultracentrifuged cleared lysates was determined using the DC method (Bio-Rad, Milano, Italy).

### 2.5. Western Immunoblot Analysis

ATGL, HSL, and phosphorylated HSL (Ser^563^) levels were detected by western blot analysis (all antibodies from Cell Signaling Technology Inc, Beverly, MA, USA). Succinate dehydrogenase complex, subunit A (SDHA) and mitochondrially-encoded cytochrome c oxidase I (mt-COX-1) levels were detected using the MitoBiogenesis™ Western Blot Cocktail (MitoSciences, Abcam, Cambridge, UK). Carbonylated proteins were measured using the OxyBlot protein oxidation detection kit (Chemicon, Billerica, MA, USA). Protein representation was quantified by densitometry (QuantityOne, BioRad), and normalized based on β-actin (Sigma-Aldrich) that was used as loading control.

### 2.6. Determination of Glycerol Release

Adipocytes were isolated from 2 portions of 1 g of minced fresh VAT, each suspended in 2.5 mL of Krebs Ringer solution containing 4% *w*/*v* BSA and 6 mM glucose (KRBAG buffer) supplemented with 3 mg/g tissue of collagenase A (Sigma Aldrich), and incubated for 1 h under shaking, at 37 °C. Minced tissue was filtered through a nylon mesh (pore size: 250 µm), then the two portions were pooled, and the suspension was centrifuged at 400 × *g*, for 1 min. The stromal cell pellet and infranatant buffer were discarded, and the cells were washed three times in 2.5 mL KRBAG buffer to remove collagenase residue. 1 × 10^6^ adipocytes from each treatment were then suspended in 2.5 mL KRBAG, and incubated in Poly-Q vials for 2 h, at 37 °C, in a 5% CO_2_ atmosphere. Hundred µL of 30% trichloroacetic acid (TCA) were added to 1 mL of the adipocyte suspension under vigorous agitation, followed by a 10 min incubation on ice and centrifugation at 1000 × *g*, for 5 min. Five hundred µL of the supernatant was neutralized with 60 µL of 10% KOH. The neutralized solution was conserved at −20 °C. An aliquot (20 µL) was then analyzed for glycerol content using the Free Glycerol Kit (Sigma Aldrich). 

### 2.7. Protein Extraction and Sample Preparation for Two-Dimensional Gel Electrophoresis (2D-E) 

2D-E was performed essentially as previously reported [40]. Briefly, tissue fragments of VAT were homogenized in a buffer containing 7 M urea, 2 M thiourea, 2% *w*/*v* CHAPS, 65 mM DTT, 0.5% *v*/*v* ampholytes, 10 mM orthovanadate and a cocktail of protease inhibitors. Proteins were extracted for 30 min, at 4 °C, and lysates were clarified by centrifugation at 20,000 × *g*, for 30 min, at 4 °C. The interface between the low-density lipid layer and the insoluble pellet was carefully collected and spun down again. Then, protein content was quantified using the DC method (Bio-Rad). Protein extracts were prepared for each animal, and each one was assayed separately. Protein samples (650 µg) were applied to immobilized pH 3–10 non-linear gradient strips (17 cm) (Bio-Rad). Focusing started at 200 V using a PROTEAN IEF System (Bio-Rad), with the voltage being gradually increased to 3500 V, and then kept constant for a further 66,500 Vh. Prior to sodium dodecyl sulphate—polyAcrylamide gel electrophoresis (SDS-PAGE), strips were equilibrated in 375 mM Tris-HCl, pH 6.8, 6 M urea, 20% *v*/*v* glycerol, 2% *w*/*v* SDS and 130 mM dithiothreitol (DTT), for 15 min, and then in the same solution containing 135 mM iodoacetamide instead of DTT, for further 15 min. The second-dimensional separation was performed by using 12% T SDS polyacrylamide gels. Protein spots were stained using colloidal Coomassie blue (according to the manufacturer’s instructions).

### 2.8. Protein Visualization and Image Analysis 

Electronic images of the gels were acquired by means of a GS-800 calibrated densitometer (Bio-Rad), and analyzed using PDQuest software (Bio-Rad). Scanned gel-images were processed for the removal of background and automatic detection of spots. For all spot-intensity calculations, normalized values were used to calculate the relative intensity (RI) for each spot: RI = vi/tid, where vi is the volume of the individual spot, and tid is the total density of the image of the gel. For each match set analysis, maps corresponding to protein extracts from animals of the same experimental group were organized into “Replicate Groups” (each containing 4 maps), named N, HFD, or HFD-T2. This allowed us to carry out a statistical analysis (Student’s t test, in pairwise comparisons) of the experimental data relative to normalized spot densities. Spots for which the P value was less than 0.05 and with at least a 2-fold variation in pairwise comparisons between selected experimental groups were considered to display a significant difference.

### 2.9. Protein Identification

Protein spots from 2D-E were excised from gels, processed, digested with trypsin and desalted with µZipTip C18 devices prior to nano-liquid chromatography (nanoLC)-electrospray ionization (ESI)-linear ion trap (LIT)-tandem (MS/MS) mass spectrometry analysis [41]. The latter was performed with a LTQ XL mass spectrometer, equipped with a Proxeon nanospray source and connected to an Easy-nanoLC (Thermo Fischer Scientific, Waltham, MA, USA). Peptide separation was achieved using an Easy C18 column (100 × 0.075 mm, 3µm) (Thermo, Waltham, MA, USA) at a flow rate of 300 nL/min, and 0.1% formic acid in water (solvent A) and 0.1% formic acid in acetonitrile (solvent B) as eluents. Eluent gradient consisted in the following steps: 5–35% B over 10 min, 35–95% B over 2 min, 95% B for 12 min. MS data were searched for protein identification through MASCOT software v2.4.2 (Matrix Science, London, UK), using a UniProtKB database of *Rattus norvegicus* (Berkenhout, 1769) protein sequences (2017_06). Searching parameters for protein identification were: mass tolerance values 2 Da for precursor ion and 0.8 Da for MS/MS fragments, trypsin as proteolytic enzyme, a missed-cleavages maximum value of 2, Cys carbamidomethylation and Met oxidation as fixed and variable modifications, respectively. Acceptance criteria for protein identification were at least 2 significant peptides (*p* < 0.05) with an individual MASCOT score > 30. Identified proteins were further evaluated by comparison of their experimental (2D-E) mass and pI values, with corresponding theoretical values. Proteins were definitively assigned when the observed emPAI^1st^ to emPAI^2nd^ ratio was > 2.0.

### 2.10. In Silico Biological Analysis

The lists of differentially expressed genes and represented proteins were input into the IPA platform (Ingenuity^®^ Systems, www.ingenuity.com) for the identification of function and canonical pathways differing between the treatments. The cut-off values used were 1.5 and 0.05 for the fold change and *p*-values, respectively. In addition, the Ingenuity Pathways Knowledge Base (IPKB) was used to analyze the whole list of differentially represented proteins in the three conditions, in terms of molecular interrelations (networks) based on their connectivity. To build networks, the program utilized was the IPKB, containing large numbers of individually modeled relationships between genes (obtained from the literature). The algorithm then determined a statistical score for each network. This was done by comparing the number of focus genes that contributed to a given network relative to the total number of occurrences of those genes in all networks or pathways stored in the IPKB. Then a score was assigned to each network. The score was the negative log of *p*, and it denoted the likelihood that the focus genes in the network might be found together by chance. Therefore, scores of 2 had an at least 99% confidence of not being generated by chance alone. In addition, the biological functions assigned to each network were ranked according to the significance of that biological function to the network. A Fisher’s exact test was used to calculate *p*, indicating the probability that the assignment of the biological function to that network might be explained by chance alone. 

### 2.11. Statistical Analysis

Data were expressed as mean values ± SEM. With exception of data obtained with the IPA platform (analyzed by a Fisher’s exact test), statistical differences of normally distributed data between 2 treatments were determined by using an unpaired Student’s t test or, for more experimental conditions, by 1-way ANOVA (post hoc test: Student-Newman-Keuls); calculations were performed with Prism 5.0 software (GraphPad Software, La Jolla, CA, USA). Differences were considered significant at a value of *p* < 0.05.

## 3. Results

### 3.1. Time Course Effect of Administration of 3,5-T2 to Rats Simultaneously Receiving HFD up to 2 Weeks on Body Weight Gain and Visceral Adiposity

A time-course study was performed to assess the early events underlying the protective effect of 3,5-T2 against fat overload in VAT. Simultaneous administration of 3,5-T2 and HFD for 1 day and 1 week (HFD-T2 groups) did not significantly change body weight and visceral fat pad weight compared to that of the HFD group (Table 1). After 1week, visceral fat pad weight tended to increase with respect to N controls both in HFD and HFD-T2 groups. After 2 weeks, HFD treatment caused a significant gain in body weight (+12% vs. N) and a non-significant increase in visceral fat pad weight (Table 1). At this time-point, the body weight of HFD-T2 group was normalized to control levels and there was a tendency toward normalization of visceral fat pad weight to control values (Table 1).

**Figure 1:**
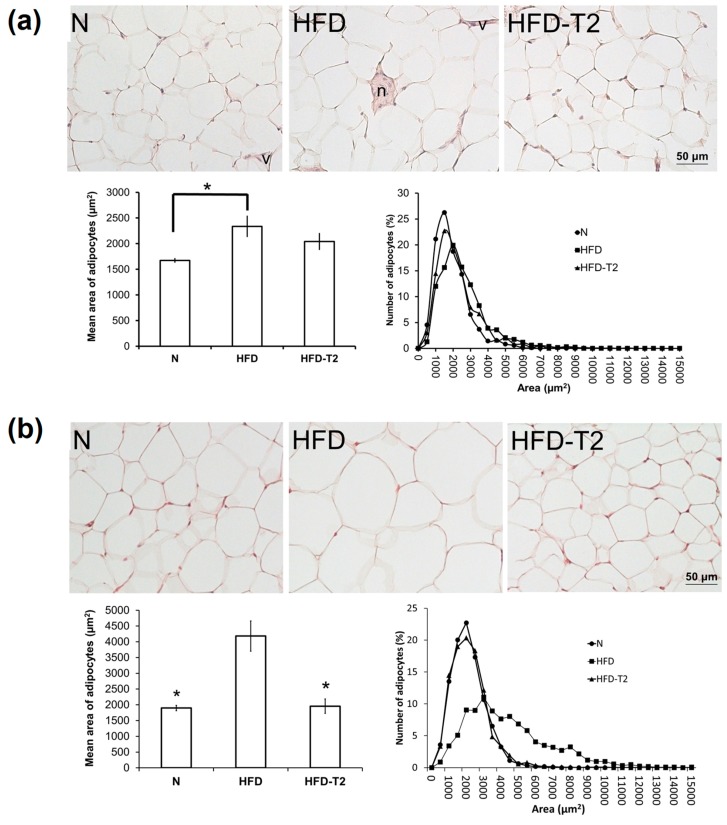
Histological and morphometrical analysis of VAT after administration of 3,5-T2 to rats simultaneously fed a high-fat diet (HFD-T2) for 1-week (**a**), and for 2 weeks (**b**), when compared to the HFD-fed (HFD) rats and chow-fed (N) controls. Representative staining of 4 independent treatments are shown. Abbreviations: *n* = nerve, *v* = vessel. Shown are the mean values ± SEM (*n* = 4). * *p* < 0.05 vs. HFD.

After 1-week of treatment, the mean surface area of the visceral adipocytes was greater in the HFD and HFD-T2 groups, reaching statistical significance in HFD with respect to N (Figure 1a; the mean area was 2040.88 ± 166 μm^2^ (HFD-T2), 2335.73 ± 207.98 μm^2^ (HFD) and 1672.50 ± 77.4 μm^2^ (N)). After 2-weeks of treatment, despite the lack of significant changes in the visceral adipocyte mass, HFD feeding substantially increased the mean surface area of the adipocytes (by 2.2-fold compared with N controls on chow diet, and histological analysis of cell distribution revealed a greater frequency of large adipocytes) (Figure 1b). At the same time point, the mean adipocyte area in the HFD-T2 group was not different from that of the N group (the mean area was 1957.35 ± 228.88 μm^2^ (HFD-T2), 4183.23 ± 481.05 μm^2^ (HFD) and 1897.85 ± 87 μm^2^ (N)).

### 3.2. Time Course Effect of Administration of 3,5-T2 to Rats Simultaneously Receiving HFD up to 2 Weeks on HSL Activation and ATGL Expression 

**Figure 2:**
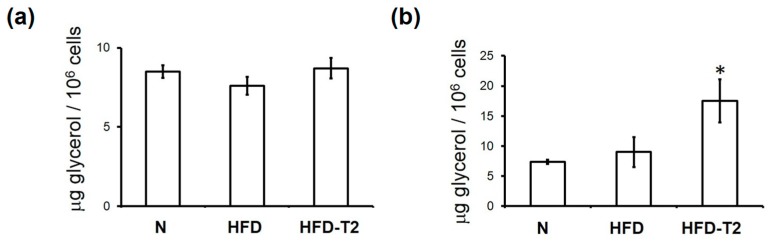
Effect of 3,5-T2 administration to rats fed a high-fat diet on the onset of lipolysis in VAT. Glycerol release from adipocytes isolated from VAT after administration of 3,5-T2 to rats simultaneously fed a high-fat diet (HFD-T2) for 1-day (**a**), and 1-week (**b**), when compared to the HFD-fed (HFD) rats and chow-fed (N) controls. The mean values shown are ± SEM (*n* = 4). * *p* < 0.05 vs. N and HFD.

**Figure 3:**
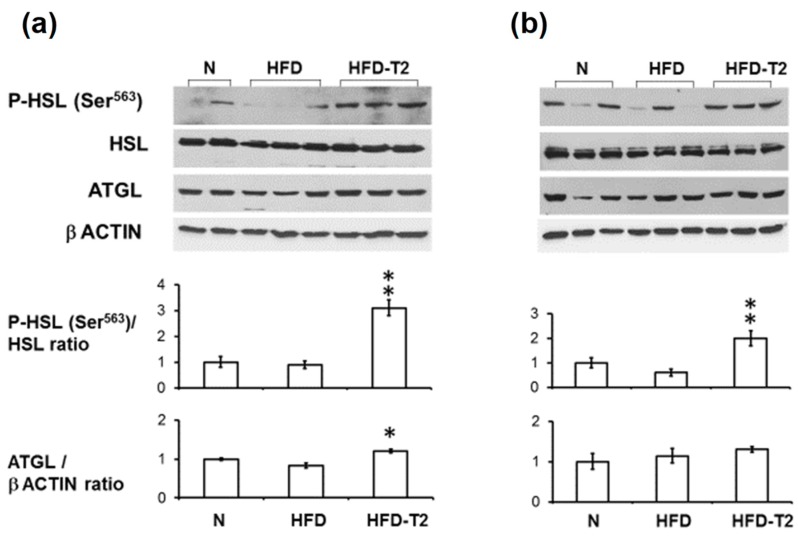
Effect of 3,5-T2 administration to rats fed a high-fat diet on signaling pathways underlying VAT lipolysis. Representative immunoblot analysis for phosphorylation and representation of hormone sensitive lipase (HSL) and adipose triglyceride lipase (ATGL) in VAT upon administration of 3,5-T2 to rats simultaneously fed a high-fat diet (HFD-T2) for 1 day (**a**), and 2 weeks (**b**). Phosphorylation of HSL at Ser^563^ was normalized with respect to total HSL. ATGL protein levels were normalized with respect to β-actin. Protein load was 30 μg/lane. Data were normalized to the value obtained for N animals (set as 1). Shown are the mean values ± SEM (*n* = 4). *p* < 0.05 * vs. HFD; ** vs. N, HFD.

Next, we investigated whether the reduction in the mean adipocyte area observed in HFD-T2 rats at the 2-week time point was preceded by a 3,5-T2-induced increase in lipolysis, measured by ex-vivo glycerol release from the VAT depots. After 1 day of treatment, no increased levels of glycerol release were observed in adipocytes isolated from the HFD-T2 group (Figure 2a); at this time-point, no differences in adipocyte volumes between the different treatment groups were also observed (data not shown). At the 1-week time-point, adipocyte volumes in HFD animals increased significantly, and T2 treatment partially normalized the adipocytes’ volume to control (N) values (Figure 1a). At this time-point a 3-fold increase of glycerol release was observed in the HFD-T2 group vs. both HFD and N group (Figure 2b). We next verified the involvement of the hormone sensitive lipase (HSL) and adipose triglyceride lipase (ATGL, also named desnutrin) in the observed lipolytic activity of 3,5-T2. After 1 day, phosphorylation of HSL at the protein kinase A (PKA) target site Ser^563^ was significantly increased in HFD-T2 with respect to the HFD group (Figure 3a). This increase persisted up to the 2-week time-point (Figure 3b). On the other hand, protein levels of the upstream lipase ATGL were significantly increased in HFD-T2 with respect to N controls after 1 day of treatment (Figure 3a) while, after 2 weeks, ATGL protein levels did not differ significantly among the groups (Figure 3b). This implies that there is an acute lipolytic stimulus governed by 3,5-T2, which may contribute to the control of adipocyte volume around the 1 week-time-point. Thereafter, peripheral fatty acid oxidation is stimulated by 3,5-T2 in such a manner that accumulation of fat in the VAT is prevented in this particular experimental set-up [18,19].

### 3.3. Effects of Administration of 3,5-T2 to Rats Simultaneously Receiving HFD up to 4 Weeks on Morphology and Vascularization of VAT 

**Figure 4:**
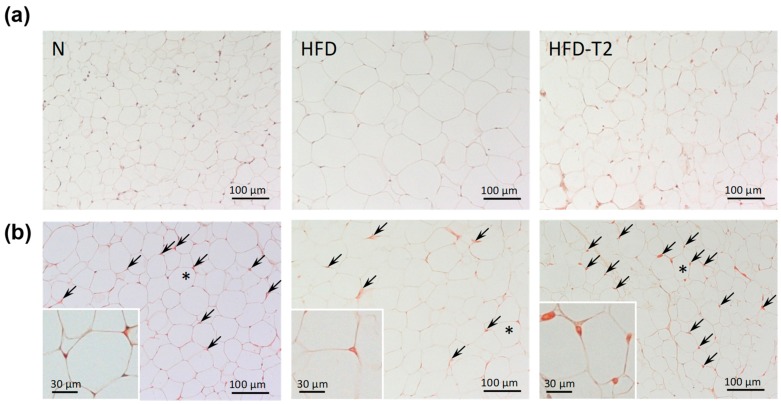
Effect of 3,5-T2 administration to rats fed a high-fat diet for 4 week on VAT morphology. (**a**) VAT was stained with hematoxylin & eosin (paraffin sections). (**b**) Vasculature was revealed by endothelial *Bandeiraea simplicifolia* agglutinin 1 (BS-1) staining (avidin-biotin complex- alkaline phosphatase (ABC-AP) method and fucsin as cromogen). BS-1-stained capillaries were indicated by arrows. Insets: high magnification of large adipocytes, as indicated by *.

As in the case of 2 weeks of treatment (as expected from our previous studies), 4 weeks of simultaneous HFD-T2 administration also produced evident reduction in adipocyte size vs. HFD (i.e., a normalization vs. N) (Figure 4a). To further characterize the structural/functional effects of 3,5-T2 on the tissue, the capillary vascularization of the VAT was detected by lectin BS-1, an immunohistochemical marker of the endothelial cells (Figure 4b). An increased vascularization was observed in VAT of HFD-T2 rats; accompanied by increased BS-1 stained capillaries in the large white adipocytes, as observed in VAT of N but not of HFD rats (Figure 4b and inserts).

### 3.4. Effect of Administration of 3,5-T2 to Rats Simultaneously Receiving HFD for 4 Weeks on the Proteomic Profile of VAT 

**Figure 5:**
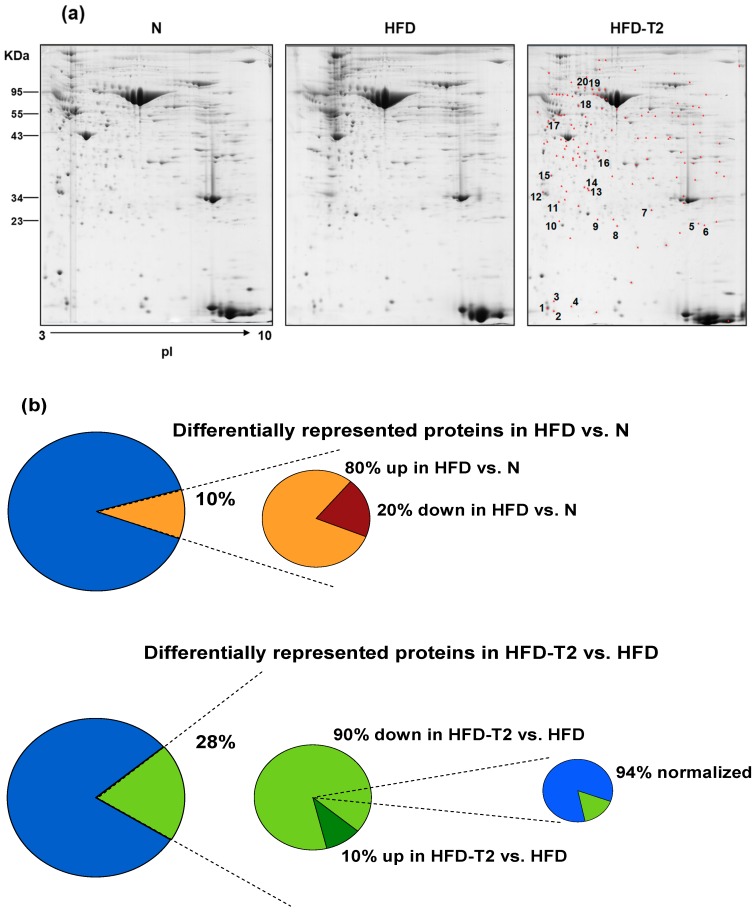
Effect of 3,5-T2 administration to rats fed a high-fat diet (HFD-T2) for 4 weeks on VAT protein repertoire. (**a**) Representative 2D-E Coomassie Blue stained maps of total VAT soluble proteins extracted from N, HFD and HFD-T2 rats. Proteins were resolved on 17 cm/pH 3–30NL IPG strips in the first dimension and on 12% T SDS-PAGE in the second one. Identified proteins among those with a density that differed significantly (by at least 0.5-or 2-fold; *p* < 0.05) between HFD-T2 and HFD (in red) are numbered. Spot numbers refer to Supplementary Tables S1 and S2. (**b**) Percentages of affected (differentially represented, over-represented, and down-represented) protein spots with respect to the total ones studied in the VAT proteome (406 protein spots) in the comparisons: HFD vs. N and HFD-T2 vs. HFD.

For each rat, the VAT was dissected, and proteins were solubilized and separated on a 2D-E gel. Overall, the 2D-E gel protein-spot patterns were qualitatively and quantitatively similar across all the gels (Figure 5a). With the detection-limits set, the software counted 450 spots per gel on average. Subsequent analysis was conducted considering only spots present under all experimental conditions. PDQuest supported pairwise comparisons between groups, detected 44 (corresponding to 10% of the total studied proteome) and 126 (corresponding to 28% of the total studied proteome) differentially represented spots (*p* < 0.05, at least 2-fold change) when comparing HFD vs. N and HFD-T2 vs. HFD, respectively (Figure 5b). Among the 44 differentially represented proteins in HFD (vs. N), 35 (80%) were over-represented and 9 (20%) were down-represented vs. N (Figure 5b). Among the 126 differentially represented proteins in HFD-T2 (vs. HFD), 14 (10%) were over-represented and 112 (90%) were down-represented vs. HFD; of these, 106 (94%) showed a normalized expression level, i.e., representation levels not significantly differing from those observed in N (Figure 5b).

Nano-LC-ESI-LIT-MS/MS analysis allowed to obtain a positive identification for 20 spots, which were associated with 17 differentially represented proteins in the comparison HFD-T2 vs. HFD (Supplementary Tables S1 and S2); the remaining spots did not provide useful data due to their low electrophoretic quality or the occurrence of multiple comigrating components (data not shown). Based on 2D-E and mass spectrometry data, it was evident that 3,5-T2-treatment significantly altered the protein representation profile displayed by the VAT depot under the HFD condition.

**Figure 6:**
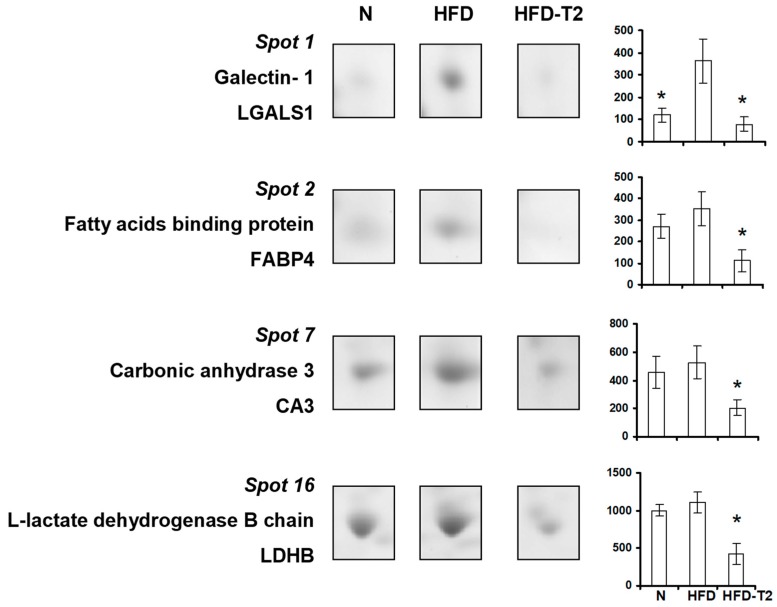
Four-week 3,5-T2 treatment-associated differential representation within the VAT proteome with regard to metabolically relevant proteins. Representative subsections of 2D-E images of VAT of N, HFD, HFD-T2 rats. Results are expressed as arbitrary units (means ± SEM; *n* = 4). * *p* < 0.05 vs. HFD. Spot numbering refers to Figure 5a.

We observed very high representation levels of galectin 1 (LGALS1) (i.e., a cellular marker of hypoxia) in VAT of HFD rats vs. both N and HFD-T2, and a normalized representation of such protein in VAT of HFD-T2 rats; this result appears coherent with the increased vascularization of the tissue despite the diet regimen in HFD-T2 rats (Figure 6).

Other VAT proteins influenced by 3,5-T2 treatment were enzymes involved in lipid and carbohydrate metabolism, namely fatty acid-binding protein (FABP4), carbonic anhydrase 3 (CA3), and L-lactate dehydrogenase B chain (LDHB). These proteins tented to be over-represented in HFD vs. N animals and were down-represented in the 2D-E maps of HFD-T2 group (Figure 6). Specifically, FABP4 is involved in enhancement of free fatty acid (FA) solubility and their storage in lipid droplets, CA3 in CO_2_ hydration and fatty acid metabolism and LDHB in generating NAD+ to convert pyruvate into lactate, Down-representation of these proteins in VAT depots of 3,5-T2-treated rats suggested a likely reduced activity of these enzymes in such animals.

**Figure 7:**
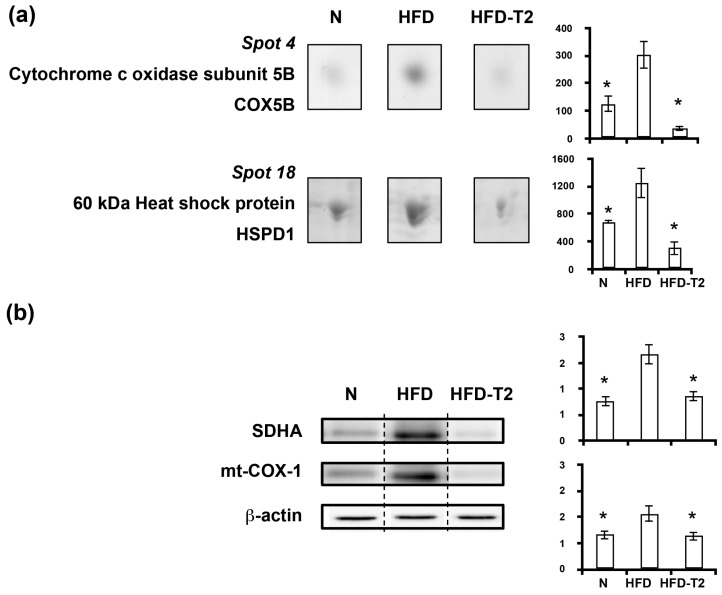
Four-week 3,5-T2 treatment-associated differential representation within the VAT proteome with regard to mitochondrial proteins. (**a**) Representative subsections of 2D-E images of VAT of N, HFD, HFD-T2 rats. Results are expressed as arbitrary units (means ± SEM; *n* = 4) (*p* < 0.05). Spot numbering refers to Figure 5a. (**b**) Representative immunoblot analysis of succinate dehydrogenase complex subunit A (SDHA) and mitochondrially-encoded cytochrome c oxidase I (mt-COX-1) protein levels in N, HFD, and HFD-T2 rats. Protein levels were normalized to β-actin. Protein load was 30 μg/lane. The vertical dotted lines indicate cuts in the membrane. Data were normalized to the value obtained for N animals (set as 1). Shown are the mean values ± SEM (*n* = 4). * *p* < 0.05 vs. HFD.

In accordance with the lipogenic phenotype of VAT of HFD fed rats was the significant over-representation of mitochondrial proteins, i.e., cytochrome c oxidase subunit 5B (COX5B) and heat shock protein 60 (HSPD1) (Figure 7a), and mitochondrial markers of biogenesis, such as succinate dehydrogenase complex subunit A (SDHA) and mitochondrially-encoded cytochrome c oxidase I (mt-COX-1) (Figure 7b). On the contrary, the anti-lipogenic action of 3,5-T2 appeared to be associated with a decreased/normalized representation of mitochondrial markers of biogenesis and of mitochondrial proteins (Figure 7).

**Figure 8:**
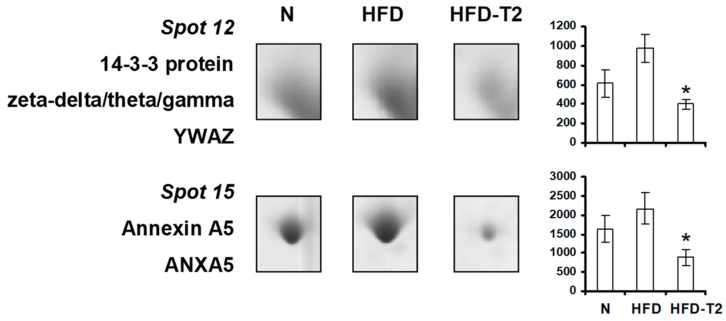
Four-week 3,5-T2 treatment-associated differential representation within the VAT proteome with regard to signaling and cellular structure proteins. Representative subsections of 2D-E images of VAT of N, HFD, HFD-T2 rats. Results are expressed as arbitrary units (means ± SEM; *n* = 4). * *p* < 0.05 vs. HFD. Spot numbering refers to Figure 5a.

A significant down-representation of 14-3-3 protein (YWAZ, 14-3-3 zeta/delta) was also observed in VAT from 3,5-T2-treated animals vs. HFD (Figure 8), further supporting an anti adipogenic/lipogenic action of 3,5-T2, if considered that 14-3-3 proteins have been recently shown to play an essential role in adipocyte differentiation both in vitro and in vivo. In parallel, 3,5-T2-treatment was associated with reduced representation levels of annexin A5 (ANXA5), a protein that, at its cellular sites (i.e., plasma membrane, nucleus, Golgi, endoplasmic reticulum, late endosomes/lysosomes, phagosomes, and mitochondria), is involved in membrane trafficking and organization, Ca^2+^ signaling and influx as well as in cell cycle regulation (Figure 8).

**Figure 9:**
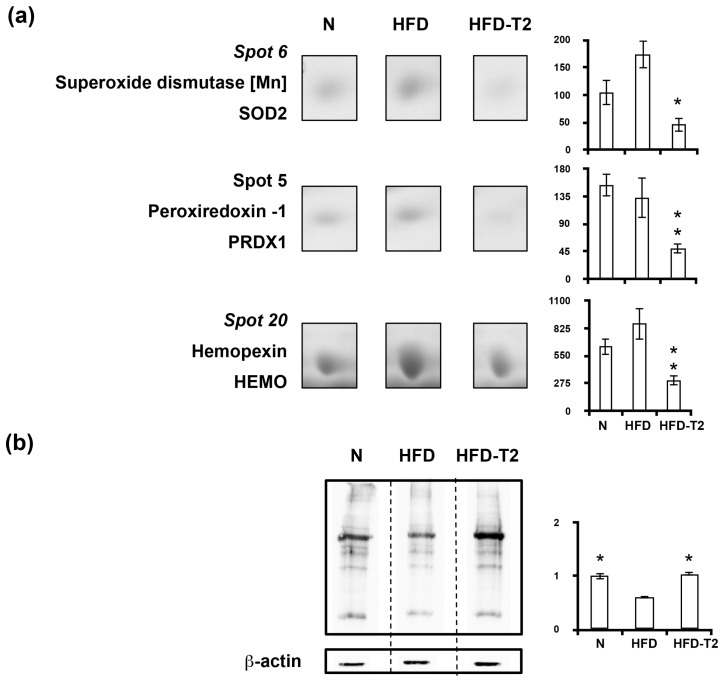
Four-week 3,5-T2 treatment-associated differential representation within the VAT proteome with regard to antioxidant proteins. (**a**) Representative subsections of 2D-E images of VAT of N, HFD, HFD-T2 rats. Results are expressed as arbitrary units (means ± SEM; *n* = 4) (*p* < 0.05). Spot numbering refers to Figure 5a. (**b**) Representative immunoblot showing protein carbonylation in VAT of N, HFD, and HFD-T2 rats. Protein load was 30 μg/lane. The vertical dotted lines indicate cuts in the membrane. Data were normalized to the value obtained for N animals (set as 1). Shown are the mean values ± SEM (*n* = 4). * *p* < 0.05 vs. HFD.

Proteomic data also positively correlated the anti adipogenic/anti lipogenic action of 3,5-T2 with decreased representation levels in VAT of antioxidant enzymes, namely superoxide dismutase (SOD2), peroxiredoxin 1 (PRDX1), and hemopexin (HEMO) (Figure 9a). These are prompts towards alterations in oxidative stress or reactive oxygen species in VAT of HFD-3,5-T2 rats, that, indeed, showed significantly higher levels of carbonylated proteins when compared to HFD rats, and a normalization of such values vs. N (Figure 9b). 

### 3.5. Canonical Pathways” and Protein Network Analysis

**Figure 10:**
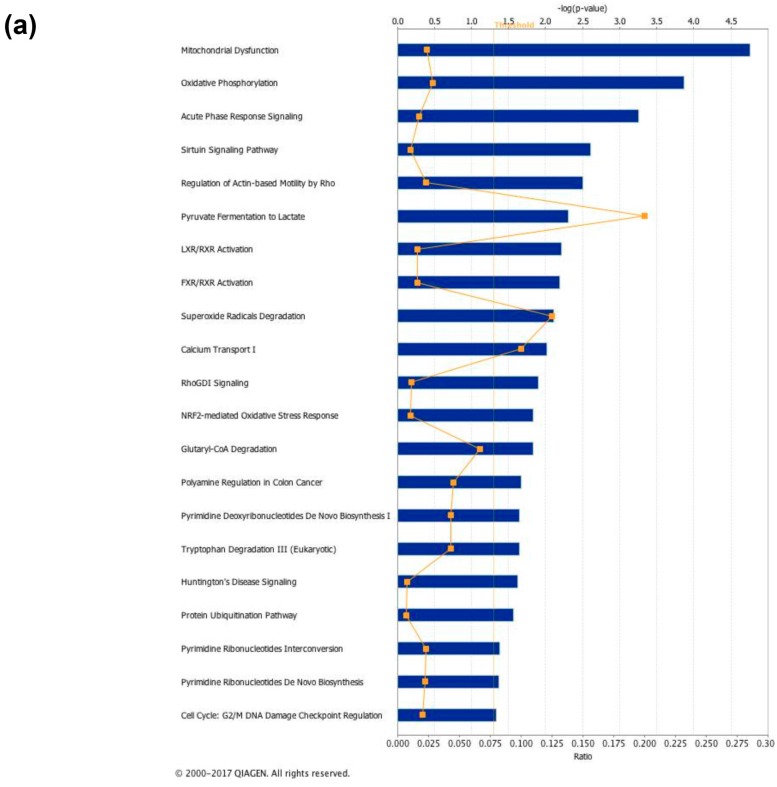

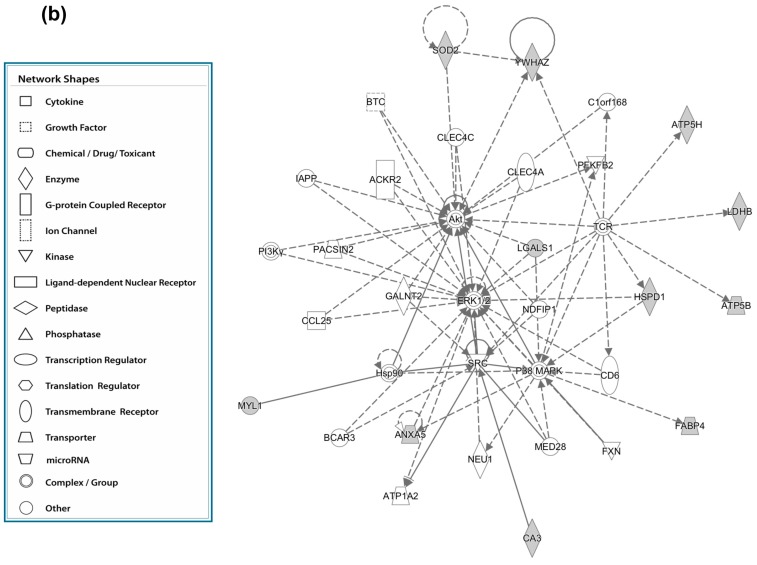
In silico analysis of canonical pathways (**a**) and protein interconnections (**b**) affected by 4 weeks of 3,5-T2 administration to high-fat fed rats. (**a**) The lists of differentially represented proteins in HFD-T2 vs HFD were input into the IPA platform (Ingenuity^®^ Systems, www.ingenuity.com) for the identification of the canonical pathways differing between the two groups. The over-represented canonical pathways for HFD-T2 vs. HFD deregulated proteins are represented in bar plots by their statistical score (negative logarithm to the base 10 of B-H corrected *p*-value) with a threshold of 1.3 (yellow straight line). The ratio between deregulated and all proteins of a pathway is also reported (yellow not straight line). (**b**) Network representation of the molecular relationships between identified, differentially represented VAT proteins in HFD-T2 vs. HFD (Ingenuity^®^ Systems Ltd.). Gene products are represented as nodes, and the biological relationship between two nodes is represented as an edge (line). Indirect interactions without physical contact appear as broken lines, whereas direct interactions requiring direct physical contact between nodes appear as solid lines. All edges shown are supported by at least 1 reference from the literature, from a textbook, or from canonical information stored in the Ingenuity Pathways Knowledge Base. Human, mouse, and rat orthologs of a gene are stored as separate objects in IPKB, but are represented as a single node in the network. For clarity, network shapes are shown. BTC = Betacellulin; C1orf168 = Chromosome 1 Open Reading Frame 168; CLEC4C = C-type lectin domain family 4 member C; CLEC4A = C-type lectin domain family 4 member A; ACKR2 = Atypical Chemokine Receptor 2; IAPP = Islet Amyloid Polypeptide; PFKFB2 = 6-phosphofructo-2-kinase/fructose-2,6-biphosphatase 2; PACSIN2 = Protein Kinase C And Casein Kinase Substrate In Neurons 2; GALNT2 = Polypeptide N-Acetylgalactosaminyltransferase 2; CCL25 = C-C Motif Chemokine Ligand 25; NDFIP1 = Nedd4 Family Interacting Protein 1; CD6 = Cluster of Differentiation 6; Hsp90 = heat shock protein 90; BCAR3 = Breast Cancer Anti-Estrogen Resistance 3; ATP1A2 = ATPase Na+/K+ Transporting Subunit Alpha 2; NEU1 = Neuraminidase 1; MED28 = Mediator Complex Subunit 28; FXN = Frataxin; PI3Kgamma = Phosphoinositide 3-kinase gamma; MAPK = Mitogen-activated protein kinase; TCR = T-cell receptor; SRC = Proto-oncogene tyrosine-protein kinase.

Analysis of the above-mentioned proteomic results by the IPA platform revealed that most significant protein representation changes induced by 3,5-T2-treatment involved canonical pathways such as, among others, mitochondrial dysfunction, oxidative phosphorylation, sirtuin signaling, pyruvate conversion to lactate, LXR/RXR and FXR/RXR activation, and superoxide radical degradation (Figure 10a).

Analysis of the molecular interrelations between differentially represented proteins generated the highest-scoring network (the score being 24) centered around two nodes, namely protein kinase B (AKT) and extracellular signal–regulated kinase (ERK)1/2 (Figure 10b), directly interconnected each other. In vitro, it has been demonstrated that AKT activity plays a crucial role in adipogenic differentiation through the parallel down-regulation of ERK1 activity, which, in contrast, sustains cell mitotic clonal expansion and thus needs to be shut off to allow adipogenesis [42]. In the obtained network, AKT is interconnected with SOD2, YWAZ and LGALS1. ERK1/2 is interconnected with ANXA5 and HSPD1 and, through Sarcoma –family kinase (SRC), with FABP4, CA3 and myosin light chain 1/3 (MYL1). Both AKT and ERK1/2 nodes projected toward the complex/group t-cell receptor (TCR), which in turn is interconnected with ATP synthase subunit d (ATP5H), ATP synthase subunit beta (ATP5B), and LDHB. The identity and the functional interrelation existing between the differential represented VAT proteins detected in the comparison HFD-T2 vs. HFD strongly supported the idea that 3,5-T2, during high-fat feeding, induces a remodeling of the VAT not only at the morphological level but also at the proteomic/metabolic one. Moreover, in silico analysis opened the way for further studies on newly unveiled iodothyronine actions.

## 4. Discussion 

Changes in adipose tissue mass and adipocyte volume have broad metabolic consequences [43]. We have previously reported that 3,5-T2 modulates VAT and prevents body-fat accumulation associated with increased hepatic fatty acid (FA) oxidation in HFD-fed rats [18,19]. Here we show that 3,5-T2 rapidly programs adipocytes for lipolysis and progressively exerts anti adipogenic/lipogenic action, which is coherent with previous data indicating that 3,5-T2 has a systemic insulin sensitizing effect [19,20,21].

At the intracellular level, lipolysis in adipose tissues is predominantly regulated by two lipases: ATGL and HSL [44]. ATGL converts triglycerides into diglycerides, supplying HSL of its preferred substrate [45,46]. Catecholamine stimulation of β-adrenergic receptors results in lipolysis through PKA-mediated phosphorylation of HSL at several targets, including Ser563 [44,47]. In agreement with this, we observed that 3,5-T2 rapidly increases HSL Ser563 phosphorylation resulting in increased glycerol efflux from adipocytes, typical for thyroid hormones, which play a permissive role on catecholamine signaling [48]. Although the lipolytic action of 3,5-T2 around the 1 week time-point induces free fatty acid (FFA) release from the tissue, the accumulation of fat in the non-adipose organs does not take place since 3,5-T2 simultaneously acts on the liver, by powerfully increasing fatty acid oxidation and decreasing lipogenesis, and significantly stimulates energy expenditure. These processes prevent fat accumulation in liver as well as in skeletal muscle [19,20]. In addition, we have previously observed in the same experimental set-up that 3,5-T2 lowered plasma nonesterified fatty acids (NEFA) levels with respect to those measured in HFD-fed animals over the entire period of treatment [19], thus supporting the notion that the lipolytic stimulus induced by 3,5-T2 occurs in concomitance with enhanced fatty acid oxidation.

At the 4-week time point, 3,5-T2-treatment prevented the HFD-induced hypertrophy and increased VAT vascularization, for the first time suggesting a proangiogenic activity of 3,5-T2 treatment, at least at the used dose, likely contributing to its insulin-sensitizing effect [19,20]. Indeed, a substantial amount of data points to a deficit in adipose tissue angiogenesis as a contributing factor to IR and metabolic disease in obesity [49,50]. Specifically, hypoperfusion can produce hypoxia, mitochondrial dysfunction and oxidative stress, which in turn may impair adipokine secretion and inflammation leading to whole-body IR [51]. It has been reported that hypoxia upregulates LGALS1 [52,53,54] and higher expression of LGALS1 has been reported in subcutaneous adipose tissue from obese subjects and diet-induced obese mice [55,56]. Accordingly, here we reported very high representation levels of LGALS1 in VAT of HFD rats vs. both N and HFD-T2, while normalized representation levels of LGALS1 in VAT of HFD-T2, paralleling the increased vascularization of the tissue despite the diet regimen in this group.

Other VAT proteins influenced by 3,5-T2-treatment resulted to be involved in lipid and carbohydrate metabolism, as well as in oxidative phosphorylation. The obtained findings were in line with the already described metabolic phenotype of 3,5-T2-treated animals characterized by ameliorated insulin sensitivity and glucose tolerance, reduced body adiposity and reduced liver steatosis despite the HFD regimen [19,20]. Indeed, in the context of dietary or genetic obesity in mice, the lack of FABP4 protects from the development of insulin resistance and type 2 diabetes, despite slightly higher body weight and elevated plasma free FA concentrations [57]. In addition, CA3 (which is downregulated by 3,5-T2) has been reported to be involved in providing bicarbonate ion to convert acetyl-CoA into malonyl-CoA necessary in lipogenesis, thus acting as a pro-lipogenic enzyme [58]. Moreover, considering that lactate is one of the main substrate for gluconeogenesis [59] and lipogenesis [60] in the liver, reduced LDHB content in VAT of 3,5-T2-treated rats not only suggested a reduced release of lactate from VAT of such animals, but was also in line with previously reported ameliorated glucose tolerance and liver steatosis in HFD-T2 rats vs. HFD ones [18,19], as well as with a lack of tissue hypertrophy and hypoxia in 3,5-T2-treated animals despite the HFD regimen. 

The anti-lipogenic action of 3,5-T2 was also associated with a decreased/normalized representation of mitochondrial proteins. Indeed, it is known that mitochondria, by generating ATP, support the highly energy-consuming lipogenic processes, to provide acetyl-CoA as a substrate for FA synthesis, and to favor the packaging of lipids in the form of triglycerides into the lipid droplet. Notably, the decreased representation of mitochondrial proteins in the VAT of 3,5-T2-treated animals may also be correlated with the decreased representation level of LDHB, when considering that lactate dehydrogenase activity and lactate production may control the expression of several proteins involved in mitochondrial activity and biogenesis [61,62].

A role for 14-3-3 proteins has been hypothesized in temporal and special control of protein–protein and protein–DNA interactions driving adipogenic factors [63]. Elevations in 14-3-3 protein levels have been reported in adipose tissue from obese individuals [64]. In mice, 14-3-3z deletion causes a marked reduction in adipose tissues, while its overexpression promotes fat tissue expansion [63]. Thus, the significant down-representation of YWAZ observed in VAT from 3,5-T2-treated animals vs. HFD, further supports an anti adipogenic/lipogenic action of 3,5-T2. 

On the other hand, annexins are calcium-dependent phospholipid binding proteins that may regulate various signaling pathways [65,66,67,68,69,70,71,72]. The repressing impact of 3,5-T2 treatment on such a protein seems coherent with the previously reported effect of the iodothyronine on cellular calcium homeostasis [73,74]. 

A large body of evidence suggests that alterations in the balance between pro oxidants and anti oxidants in adipose tissue not only correlates with IR but is also causative of its development [75]. Data obtained here positively correlated the anti adipogenic/anti lipogenic action of 3,5-T2 with decreased representation levels in VAT of antioxidant enzymes and higher levels of carbonylated proteins (when compared to HFD rats). Adipose-specific deletion of SOD2 has been reported to be protective against HFD-induced weight gain and IR [76]; moreover, it has been suggested that ROS have also been involved in pathways of adipocyte apoptosis [77]. Thus, modulation of antioxidant enzymes as well as of cellular ROS levels may be a part of the signaling pathways through which 3,5-T2 inhibits fat tissue accumulation under HFD conditions.

## 5. Conclusions

Taken together, our analysis provided novel findings showing how 3,5-T2, when simultaneously administered to rats exposed to HFD, rapidly (within 1 day after administration) promotes visceral adipose lipolysis through HSL activation. Long-term treatment with 3,5-T2 produced effects on adipocyte morphology (already measurable after 2 weeks and persistent at 4 weeks of treatment), tissue vascularization, and the protein profile. 3,5-T2 effects on 2D-E-based proteomic maps involved about 30% of the resolved components. After 4 weeks of treatment, clear-cut effects of HFD and 3,5-T2 treatment were measured on markers of hypoxia, mitochondrial biogenesis and oxidative stress. These features, combined with the increased oxidative capacity of liver we described in previous studies, expand on the notion of the prevention of fat mass-gain by 3,5-T2, when administered during high-fat feeding in the rat, and contribute to explaining the action of this intriguing thyroid hormone metabolite. Although the physiological relevance of 3,5-T2 is still unclear, pharmacological and studies performed over the last 20 years have furnished an increasing amount of information on its cellular targets and affected pathways. One limitation of this study is that only a single type of HFD is studied, namely the classic “western cafè diet”. The dietary context is a determining factor for the action of 3,5-T2 in liver [32], muscle [78], and presumably also in adipose tissue; at least in part, it is related to the saturation degree of fatty acids [78], which is a key feature to be explored further in future studies. It is important to further consider the eventual application of this thyroid hormone metabolite in a clinical setting. 

## Figures and Tables

**Table 1:** Body weight and visceral adipose tissue mass of animals treated as indicated ^1^.

	1 DAY			1 WEEK			2 WEEKS		
	N	HFD	HFD-T2	N	HFD	HFD-T2	N	HFD	HFD-T2
**% BW**	100 ± 3	99 ± 5	102 ± 3	100 ± 7	107 ± 2	106 ± 3	100 ± 3	112 ± 3 *	106 ± 2
**WW (g)**	11.3 ± 1.5	11.8 ± 1.3	12.9 ± 0.4	11.9 ± 0.7	14.5 ± 1.6	15.1 ± 0.8	13.3 ± 1.2	22.8 ± 4.8	18.9 ± 5.6
**% adip.**	2.7 ± 0.3	2.8 ± 0.2	3.0 ± 0.2	2.7 ± 0.1	3.0 ± 0.3	3.2 ± 0.2	3.3 ± 0.2	5.0 ± 0.9	4.4 ± 1.0

^1^ Abbreviations: % BW = % body weight of treated animals vs. controls (N) set as 100%; WW = visceral white adipose tissue weight; % adip. = adiposity; N = chow-fed control animals; HFD = high-fat diet fed animals; HFD-T2 = HFD and 3,5-T2-treated animals. Shown are the mean values ± SEM of 4 independent treatments. *p* < 0.05 * vs. N, HFD-T2.

## Supplementary Materials

The supplementary materials can be found at http://www.mdpi.com/2072-6643/11/2/278/s1. Table S1: List of differentially represented proteins identified in this study. VAT proteins were extracted, separated by 2D-E, digested *in-gel* with trypsin and identified by nanoLC-ESI-LIT-MS/MS. Bioinformatics was performed as defined in the experimental section; at least 2 matching peptides were required for identity assignment. Spot numbering corresponds to that indicated in Figure 5A. Abbreviation and UniProtKB accession numbers of the identified proteins are reported, together with identified peptides, sequence coverage (%), theoretical Mr and pI values and sequence identity of the UniProtKB sequence entry with respect to the NCBI one (%), Table S2: Identification details for the protein entries reported in Supplementary Table S1.
